# (*R*)-Doxylaminium (*R*,*R*)-tartrate

**DOI:** 10.1107/S160053681200935X

**Published:** 2012-03-14

**Authors:** A.S. Dayananda, Grzegorz Dutkiewicz, H. S. Yathirajan, Maciej Kubicki

**Affiliations:** a Department of Studies in Chemistry, University of Mysore, Mysore 570 006, India; b Department of Chemistry, Adam Mickiewicz University, Grunwaldzka 6, 60-780 Poznań, Poland

## Abstract

In the title compound (systematic name: (*R*)-dimeth­yl{2-[1-phenyl-1-(pyridin-2-yl)eth­oxy]eth­yl}aza­nium (*R*,*R*)-3-carb­oxy-2,3-dihy­droxy­propano­ate), C_17_H_23_N_2_O^+^·C_4_H_5_O_6_^−^, the doxylaminium cation is protonated at the N atom. The tartrate monoanions are linked by short, almost linear O—H⋯O hydrogen bonds into chains extended along [100]. These chains are inter­linked by anion–pyridine O—H⋯N hydrogen bonds into a two-dimensional grid structure. WeakC—H⋯O inter­actions also play a role in the crystal packing. An intra­molecular hy­droxy–carboxyl­ate O—H⋯O hydrogen bond influences the conformation of the anion: the hydrogen-bonded fragment is almost planar, the maximum deviation from the mean plane being 0.059 (14) Å. In the cation, the aromatic rings are almost perpendicular [dihedral angle = 84.94 (8)°] and the conformation of the O—C—C—N chain is *gauche*(−), the dihedral angle is −76.6 (2)°. The absolute configuration was assigned on the basis of known chirality of the parent compound.

## Related literature

For related strucures, see: Braitenbach & Parvez (2001 ▶); Parvez & Sabir (1998 ▶); Parvez *et al.* (2001 ▶). For general literature on doxyl­amine, see, for example: Casy (1991 ▶); Eccles *et al.* (1995 ▶). For graph-set motifs, see: Etter *et al.* (1990 ▶); Bernstein *et al.* (1995 ▶). For a description of the Cambridge Structural Database, see: Allen (2002 ▶).
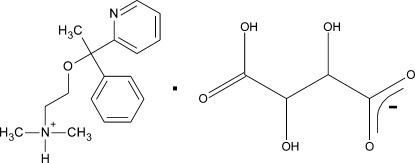


## Experimental

### 

#### Crystal data

C_17_H_23_N_2_O^+^·C_4_H_5_O_6_^−^
*M* *_r_* = 420.45
Monoclinic, 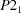

*a* = 7.4419 (4) Å
*b* = 18.4394 (8) Å
*c* = 8.3517 (4) Å
β = 108.301 (5)°
*V* = 1088.09 (9) Å^3^
*Z* = 2
Mo *K*α radiation
μ = 0.10 mm^−1^
*T* = 295 K
0.35 × 0.2 × 0.15 mm

#### Data collection

Agilent Xcalibur Eos diffractometer
Absorption correction: multi-scan (*CrysAlis PRO*; Agilent, 2011 ▶) *T*_min_ = 0.991, *T*_max_ = 1.000
4562 measured reflections
3539 independent reflections
3228 reflections with *I* > 2σ(*I*)
*R*_int_ = 0.011

#### Refinement

*R*[*F* ^2^ > 2σ(*F* ^2^)] = 0.035
*wR*(*F* ^2^) = 0.079
*S* = 1.06
3539 reflections
290 parameters
1 restraint
H atoms treated by a mixture of independent and constrained refinement
Δρ_max_ = 0.11 e Å^−3^
Δρ_min_ = −0.16 e Å^−3^

### 

Data collection: *CrysAlis PRO* (Agilent, 2011 ▶); cell refinement: *CrysAlis PRO*; data reduction: *CrysAlis PRO*; program(s) used to solve structure: *SIR92* (Altomare *et al.*, 1993 ▶); program(s) used to refine structure: *SHELXL97* (Sheldrick, 2008 ▶); molecular graphics: *XP* (Sheldrick, 2008 ▶); software used to prepare material for publication: *SHELXL97*.

## Supplementary Material

Crystal structure: contains datablock(s) I, global. DOI: 10.1107/S160053681200935X/nr2021sup1.cif

Structure factors: contains datablock(s) I. DOI: 10.1107/S160053681200935X/nr2021Isup2.hkl

Supplementary material file. DOI: 10.1107/S160053681200935X/nr2021Isup3.cml

Additional supplementary materials:  crystallographic information; 3D view; checkCIF report

## Figures and Tables

**Table 1:** Hydrogen-bond geometry (Å, °)

*D*—H⋯*A*	*D*—H	H⋯*A*	*D*⋯*A*	*D*—H⋯*A*
C42—H42*A*⋯O3*A*	0.97	2.52	3.391 (3)	149
C43—H43*B*⋯O41*A*^i^	0.97	2.27	3.217 (3)	165
N44—H44⋯O12*A*	0.86 (3)	2.23 (3)	2.942 (2)	140 (2)
O2*A*—H2*A*1⋯N32^ii^	0.89 (4)	1.92 (4)	2.804 (2)	171 (3)
O3*A*—H3*A*1⋯O41*A*	0.81 (3)	2.08 (3)	2.613 (2)	123 (3)
O42*A*—H42⋯O12*A*^iii^	1.12 (3)	1.33 (3)	2.4475 (18)	178 (3)

Symmetry codes: (i) 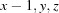; (ii) 
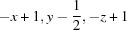
; (iii) 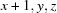.

**Fig. 1.**
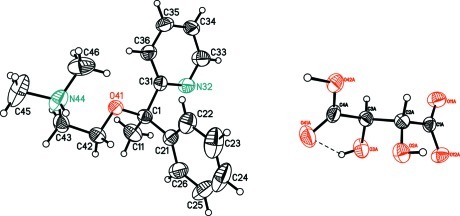
Anisotropic ellipsoid representation of the ionic components of the salt 1, together with atom labelling scheme. The ellipsoids are drawn at 50% probability level, hydrogen atoms are depicted as spheres with arbitrary radii. Intramolecular hydrogen bond is shown as dashed line.

**Fig. 2.**
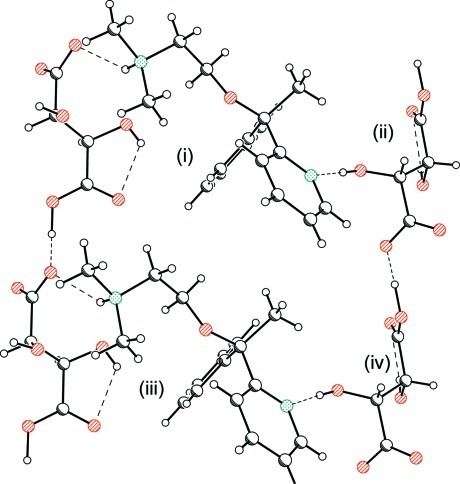
The hydrogen-bonded ring of cations and anions. Hydrogen bonds are drawn as dashed lines, symmetry codes: (i) x,y,z; (ii) 1 - x,1/2 + y,1 - z; (iii) -1 + x,y,z; (iv) -x,1/2 + y,1 - z.

**Fig. 3.**
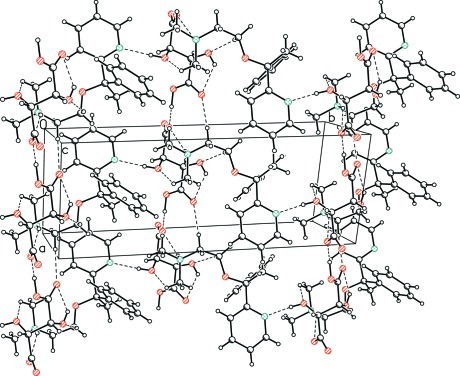
The crystal packing as seen approximately along c-direction, hydrogen bonds, including weak C—H···O interactions listed in table 1) are drawn as dashed lines.

## supplementary crystallographic information

### Comment

Doxylamine (dimethyl-[2-(1-phenyl-1-pyridin-2-yl-ethoxy)-ethyl]-amine) is a chiral tertiary aminoalkyl ether, which exhibits an antihistaminic action on the H_1_ receptor site (*e.g.* Casy, 1991). It is used as a short-term sedative, and - in combination with other drugs - as a night-time cold and allergy relief drug (Eccles *et al.*, 1995).

There are not many crystal structures of related compounds in the Cambridge Crystallographic Database (Allen, 2002), and these are exclusively salts: monoprotonated doxylaminium hydrogen succinate (Parvez *et al.*, 2001), and diprotonated doxylaminium tetrachlorocuprate(II) (Braitenbach & Parvez, 2001), and isostructural tetrachlorozincate(II) and tetrachlorocobaltate(II) (Parvez & Sabir, 1998). In view of the importance of doxylamine, we have determined the crystal and molecular structure of the title salt, (1, Scheme 1), (*R*)-doxylaminium (*R*,*R*)-tartarate. The absolute configuration was assigned on the basis of known chirality of the parent compound.

In 1 doxylamine is monoprotonated at N44, thus giving a quaternary ammonium cation (Fig. 1). This 'additional' hydrogen atom was found in the difference Fourier map and successfully refined. The aromatic rings in the cation are almost perpendicular, the dihedral angle between the least-squares planes of phenyl (planar within 0.0064 (16) Å) and pyridine (0.0056 (16) Å) rings is 84.94 (8)°. The conformation along C—O—C—C—N chain is *tg^-^*, the appropriate torsion angles are -164.77 (18)° and -76.6 (2)°. Similar conformation has been observed in previously reported doxylamine salts, despite the presence of intra-cationic hydrogen bonds in the di-cations (Parvez & Sabir, 1998, Braitenbach & Parvez, 2001).

In the anion the carbon chain is in an extended conformation (C—C—C—C torsion angle is -178.33 (16)°), and the overall conformation of the anion might be described by the dihedral angle betwen the two approximately planar 'halves': C1A, O11A, O12A, C2A and C3A, C4A, O41A, O42A, which is 43.45 (8) °. It might be noted that due to the intramolecular O3A—H···O41A hydrogen bond (*cf.* Table 1), the O3A oxygen atom is almost coplanar with the CCOO plane (0.033 (4) Å), while O2A is significantly deviated from similar plane, by -0.420 (3) Å.

In the crystal very short and almost linear O—H···O (*x* + 1,*y*,*z*) hydrogen bonds (O—H 1.12 (3) Å, H···O 1.33 (3) Å, O···O 2.4475 (18) Å, O—H···O 178 (3) °) connect the anions into infinite chains along *x* direction. As frequent happens for this kind of short bonds, the O—H bond is elongated, while H···O contact is quite short, which might suggest the covalent component in both of them. The anionic chains are connexted with the cations by means of N—H···O and O—H···N hydrogen bonds creating the rings (Fig. 2) which can be described as *R*^6^_5_(36) (Etter *et al.*, 1990, Bernstein *et al.*, 1995). Repetition of these rings produces the chessboard-like pattern of anions and cations in the crystal structure (Fig. 3). Some secondary C—H···O interactions are also playing a role in the crystal packing.

### Experimental

The title compound was obtained as a gift sample from *R. L*. Fine Chem., Bengaluru, India. The compound was recrystallized from methanol by slow evaporation (m.p: 388 K).

### Refinement

Hydrogen atoms attached to O and N atoms were found in difference Fourier maps and isotropically refined, all other H atoms were put in the idealized positions, and refined as riding model. Their isotropic thermal parameters were set at 1.2 (1.5 for methyl groups) times *U*_eq_'s of appropriate carrier atoms. The Friedel pairs were not merged, however their coverage is relatively low, of *ca* 50%.

### Crystal data

**Table:**

C_17_H_23_N_2_O^+^·C_4_H_5_O_6_^−^	*F*(000) = 448
*M**_r_* = 420.45	*D*_x_ = 1.283 Mg m^−^^3^
Monoclinic, *P*2_1_	Mo *K*α radiation, λ = 0.7107 Å
*a* = 7.4419 (4) Å	Cell parameters from 1885 reflections
*b* = 18.4394 (8) Å	θ = 2.9–27.8°
*c* = 8.3517 (4) Å	µ = 0.10 mm^−^^1^
β = 108.301 (5)°	*T* = 295 K
*V* = 1088.09 (9) Å^3^	Block, colourless
*Z* = 2	0.35 × 0.2 × 0.15 mm

### Data collection

**Table:**

Agilent Xcalibur Eos diffractometer	3539 independent reflections
Radiation source: Enhance (Mo) X-ray Source	3228 reflections with *I* > 2σ(*I*)
Graphite monochromator	*R*_int_ = 0.011
Detector resolution: 16.1544 pixels mm^-1^	θ_max_ = 27.9°, θ_min_ = 2.9°
ω scan	*h* = −5→9
Absorption correction: multi-scan (*CrysAlis PRO*; Agilent, 2011)	*k* = −23→19
*T*_min_ = 0.991, *T*_max_ = 1.000	*l* = −10→10
4562 measured reflections	

### Refinement

**Table:**

Refinement on *F*^2^	Primary atom site location: structure-invariant direct methods
Least-squares matrix: full	Secondary atom site location: difference Fourier map
*R*[*F*^2^ > 2σ(*F*^2^)] = 0.035	Hydrogen site location: inferred from neighbouring sites
*wR*(*F*^2^) = 0.079	H atoms treated by a mixture of independent and constrained refinement
*S* = 1.06	*w* = 1/[σ^2^(*F*_o_^2^) + (0.0316*P*)^2^ + 0.1652*P*] where *P* = (*F*_o_^2^ + 2*F*_c_^2^)/3
3539 reflections	(Δ/σ)_max_ = 0.001
290 parameters	Δρ_max_ = 0.11 e Å^−^^3^
1 restraint	Δρ_min_ = −0.16 e Å^−^^3^

### Special details

**Table:**

Geometry. All s.u.'s (except the s.u. in the dihedral angle between two l.s. planes) are estimated using the full covariance matrix. The cell s.u.'s are taken into account individually in the estimation of s.u.'s in distances, angles and torsion angles; correlations between s.u.'s in cell parameters are only used when they are defined by crystal symmetry. An approximate (isotropic) treatment of cell s.u.'s is used for estimating s.u.'s involving l.s. planes.
Refinement. Refinement of *F*^2^ against ALL reflections. The weighted *R*-factor *wR* and goodness of fit *S* are based on *F*^2^, conventional *R*-factors *R* are based on *F*, with *F* set to zero for negative *F*^2^. The threshold expression of *F*^2^ > σ(*F*^2^) is used only for calculating *R*-factors(gt) *etc*. and is not relevant to the choice of reflections for refinement. *R*-factors based on *F*^2^ are statistically about twice as large as those based on *F*, and *R*- factors based on ALL data will be even larger.

### Fractional atomic coordinates and isotropic or equivalent isotropic displacement parameters (Å^2^)

**Table:**

	*x*	*y*	*z*	*U*_iso_*/*U*_eq_	
C1	0.4272 (3)	0.67447 (11)	0.6599 (2)	0.0349 (5)	
C11	0.3002 (4)	0.71964 (15)	0.7339 (3)	0.0537 (6)	
H11A	0.1778	0.7251	0.6511	0.081*	
H11B	0.3559	0.7666	0.7654	0.081*	
H11C	0.2870	0.6956	0.8316	0.081*	
C21	0.4472 (3)	0.70427 (11)	0.4958 (3)	0.0372 (5)	
C22	0.5716 (4)	0.67112 (15)	0.4264 (3)	0.0524 (6)	
H22	0.6417	0.6313	0.4799	0.063*	
C23	0.5930 (5)	0.69689 (19)	0.2769 (4)	0.0790 (10)	
H23	0.6777	0.6744	0.2313	0.095*	
C24	0.4894 (6)	0.7554 (2)	0.1966 (4)	0.0884 (13)	
H24	0.5045	0.7727	0.0970	0.106*	
C25	0.3652 (6)	0.78785 (17)	0.2625 (4)	0.0791 (11)	
H25	0.2945	0.8273	0.2075	0.095*	
C26	0.3427 (4)	0.76266 (14)	0.4116 (3)	0.0562 (7)	
H26	0.2565	0.7852	0.4553	0.067*	
C31	0.6242 (3)	0.66822 (12)	0.7900 (2)	0.0345 (4)	
N32	0.7191 (3)	0.73082 (10)	0.8328 (2)	0.0462 (5)	
C33	0.8949 (4)	0.72791 (14)	0.9414 (3)	0.0574 (7)	
H33	0.9616	0.7712	0.9701	0.069*	
C34	0.9824 (3)	0.66531 (15)	1.0129 (3)	0.0504 (6)	
H34	1.1049	0.6659	1.0880	0.061*	
C35	0.8848 (4)	0.60232 (14)	0.9705 (3)	0.0536 (7)	
H35	0.9394	0.5587	1.0171	0.064*	
C36	0.7034 (3)	0.60348 (13)	0.8576 (3)	0.0477 (6)	
H36	0.6354	0.5606	0.8277	0.057*	
O41	0.3570 (2)	0.60073 (8)	0.63556 (17)	0.0390 (4)	
C42	0.1862 (3)	0.58833 (13)	0.5017 (3)	0.0426 (5)	
H42A	0.2110	0.5902	0.3945	0.051*	
H42B	0.0949	0.6258	0.5019	0.051*	
C43	0.1071 (3)	0.51528 (13)	0.5234 (3)	0.0464 (6)	
H43A	0.1131	0.5100	0.6405	0.056*	
H43B	−0.0253	0.5141	0.4559	0.056*	
N44	0.2040 (3)	0.45177 (11)	0.4760 (2)	0.0503 (5)	
H44	0.193 (4)	0.4545 (15)	0.371 (3)	0.057 (7)*	
C45	0.1053 (6)	0.38380 (16)	0.4956 (4)	0.0868 (11)	
H45A	0.1210	0.3759	0.6128	0.130*	
H45B	0.1582	0.3437	0.4524	0.130*	
H45C	−0.0270	0.3879	0.4341	0.130*	
C46	0.4095 (4)	0.44705 (16)	0.5709 (3)	0.0659 (8)	
H46A	0.4265	0.4436	0.6894	0.099*	
H46B	0.4725	0.4896	0.5493	0.099*	
H46C	0.4621	0.4049	0.5351	0.099*	
C1A	0.0805 (3)	0.41864 (11)	0.0025 (2)	0.0315 (4)	
O11A	−0.0017 (2)	0.42724 (10)	−0.14690 (18)	0.0513 (4)	
O12A	0.00315 (19)	0.41842 (9)	0.12021 (18)	0.0435 (4)	
C2A	0.2953 (3)	0.40945 (11)	0.0626 (2)	0.0304 (4)	
H2A	0.3307	0.3787	−0.0184	0.036*	
O2A	0.3691 (2)	0.37835 (9)	0.22459 (18)	0.0396 (4)	
H2A1	0.344 (5)	0.331 (2)	0.219 (4)	0.095 (12)*	
C3A	0.3858 (3)	0.48319 (11)	0.0658 (3)	0.0335 (4)	
H3A	0.3418	0.5043	−0.0476	0.040*	
O3A	0.3330 (2)	0.52934 (9)	0.1788 (2)	0.0472 (4)	
H3A1	0.431 (4)	0.5477 (17)	0.236 (4)	0.070 (10)*	
C4A	0.5998 (3)	0.47547 (12)	0.1202 (3)	0.0360 (5)	
O41A	0.6965 (2)	0.50948 (10)	0.2428 (2)	0.0549 (5)	
O42A	0.6590 (2)	0.43365 (9)	0.0257 (2)	0.0470 (4)	
H42	0.816 (5)	0.4273 (19)	0.067 (4)	0.095 (10)*	

### Atomic displacement parameters (Å^2^)

**Table:**

	*U*^11^	*U*^22^	*U*^33^	*U*^12^	*U*^13^	*U*^23^
C1	0.0333 (10)	0.0350 (12)	0.0343 (10)	−0.0003 (10)	0.0076 (8)	−0.0030 (9)
C11	0.0449 (13)	0.0630 (17)	0.0533 (14)	0.0032 (13)	0.0154 (11)	−0.0132 (12)
C21	0.0427 (12)	0.0327 (11)	0.0315 (10)	−0.0088 (10)	0.0047 (9)	−0.0027 (8)
C22	0.0659 (16)	0.0510 (14)	0.0456 (13)	−0.0069 (14)	0.0252 (12)	−0.0047 (11)
C23	0.110 (3)	0.082 (2)	0.0596 (18)	−0.041 (2)	0.0468 (19)	−0.0242 (17)
C24	0.135 (3)	0.084 (3)	0.0394 (15)	−0.070 (2)	0.0173 (19)	−0.0019 (16)
C25	0.104 (3)	0.0553 (19)	0.0509 (17)	−0.0315 (19)	−0.0144 (17)	0.0186 (14)
C26	0.0590 (16)	0.0426 (14)	0.0527 (14)	−0.0067 (13)	−0.0030 (12)	0.0058 (11)
C31	0.0364 (11)	0.0334 (11)	0.0343 (10)	−0.0040 (10)	0.0119 (8)	−0.0004 (9)
N32	0.0458 (11)	0.0314 (10)	0.0516 (11)	−0.0025 (9)	0.0012 (9)	−0.0018 (8)
C33	0.0480 (14)	0.0370 (13)	0.0696 (17)	−0.0080 (12)	−0.0069 (13)	−0.0025 (12)
C34	0.0364 (12)	0.0471 (14)	0.0565 (14)	−0.0046 (12)	−0.0016 (10)	0.0051 (11)
C35	0.0496 (14)	0.0397 (14)	0.0600 (15)	0.0014 (12)	0.0007 (12)	0.0134 (11)
C36	0.0441 (13)	0.0355 (13)	0.0531 (13)	−0.0067 (11)	0.0003 (11)	0.0055 (10)
O41	0.0360 (8)	0.0380 (9)	0.0358 (7)	−0.0070 (7)	0.0007 (6)	0.0027 (6)
C42	0.0364 (12)	0.0467 (13)	0.0384 (11)	−0.0038 (11)	0.0026 (9)	−0.0004 (10)
C43	0.0421 (13)	0.0539 (15)	0.0386 (12)	−0.0148 (12)	0.0060 (10)	−0.0045 (11)
N44	0.0770 (15)	0.0429 (11)	0.0271 (10)	−0.0149 (11)	0.0106 (10)	−0.0013 (8)
C45	0.159 (3)	0.0560 (17)	0.0523 (16)	−0.046 (2)	0.043 (2)	−0.0080 (13)
C46	0.082 (2)	0.0551 (16)	0.0463 (15)	0.0186 (15)	−0.0007 (14)	−0.0058 (12)
C1A	0.0257 (9)	0.0330 (11)	0.0360 (10)	−0.0023 (9)	0.0100 (8)	−0.0020 (9)
O11A	0.0340 (7)	0.0819 (13)	0.0354 (8)	0.0022 (8)	0.0070 (7)	0.0005 (8)
O12A	0.0272 (7)	0.0677 (11)	0.0392 (8)	0.0042 (7)	0.0155 (6)	0.0038 (8)
C2A	0.0249 (9)	0.0335 (11)	0.0330 (10)	0.0038 (9)	0.0098 (8)	−0.0039 (8)
O2A	0.0344 (8)	0.0369 (9)	0.0412 (8)	0.0017 (7)	0.0029 (6)	0.0025 (7)
C3A	0.0263 (10)	0.0377 (11)	0.0361 (10)	0.0010 (9)	0.0093 (8)	−0.0033 (9)
O3A	0.0347 (9)	0.0432 (10)	0.0643 (11)	0.0004 (8)	0.0161 (8)	−0.0182 (8)
C4A	0.0256 (10)	0.0416 (12)	0.0420 (11)	−0.0048 (10)	0.0125 (9)	0.0004 (10)
O41A	0.0317 (8)	0.0688 (12)	0.0575 (10)	−0.0053 (8)	0.0042 (7)	−0.0198 (9)
O42A	0.0244 (7)	0.0647 (11)	0.0551 (9)	−0.0016 (7)	0.0172 (7)	−0.0156 (8)

### Geometric parameters (Å, º)

**Table:**

C1—O41	1.448 (2)	C42—C43	1.503 (3)
C1—C21	1.526 (3)	C42—H42A	0.9700
C1—C11	1.528 (3)	C42—H42B	0.9700
C1—C31	1.531 (3)	C43—N44	1.492 (3)
C11—H11A	0.9600	C43—H43A	0.9700
C11—H11B	0.9600	C43—H43B	0.9700
C11—H11C	0.9600	N44—C45	1.487 (3)
C21—C22	1.380 (3)	N44—C46	1.488 (3)
C21—C26	1.384 (3)	N44—H44	0.86 (3)
C22—C23	1.390 (4)	C45—H45A	0.9600
C22—H22	0.9300	C45—H45B	0.9600
C23—C24	1.372 (5)	C45—H45C	0.9600
C23—H23	0.9300	C46—H46A	0.9600
C24—C25	1.356 (5)	C46—H46B	0.9600
C24—H24	0.9300	C46—H46C	0.9600
C25—C26	1.387 (4)	C1A—O11A	1.216 (2)
C25—H25	0.9300	C1A—O12A	1.285 (2)
C26—H26	0.9300	C1A—C2A	1.527 (3)
C31—N32	1.342 (3)	C2A—O2A	1.412 (2)
C31—C36	1.372 (3)	C2A—C3A	1.514 (3)
N32—C33	1.339 (3)	C2A—H2A	0.9800
C33—C34	1.367 (4)	O2A—H2A1	0.89 (4)
C33—H33	0.9300	C3A—O3A	1.415 (3)
C34—C35	1.357 (4)	C3A—C4A	1.519 (3)
C34—H34	0.9300	C3A—H3A	0.9800
C35—C36	1.383 (3)	O3A—H3A1	0.81 (3)
C35—H35	0.9300	C4A—O41A	1.223 (2)
C36—H36	0.9300	C4A—O42A	1.277 (2)
O41—C42	1.423 (2)	O42A—H42	1.12 (3)
			
O41—C1—C21	110.18 (16)	C43—C42—H42A	109.7
O41—C1—C11	109.08 (18)	O41—C42—H42B	109.7
C21—C1—C11	114.45 (19)	C43—C42—H42B	109.7
O41—C1—C31	104.58 (16)	H42A—C42—H42B	108.2
C21—C1—C31	108.82 (17)	N44—C43—C42	115.6 (2)
C11—C1—C31	109.26 (17)	N44—C43—H43A	108.4
C1—C11—H11A	109.5	C42—C43—H43A	108.4
C1—C11—H11B	109.5	N44—C43—H43B	108.4
H11A—C11—H11B	109.5	C42—C43—H43B	108.4
C1—C11—H11C	109.5	H43A—C43—H43B	107.4
H11A—C11—H11C	109.5	C45—N44—C46	110.7 (3)
H11B—C11—H11C	109.5	C45—N44—C43	109.6 (2)
C22—C21—C26	118.4 (2)	C46—N44—C43	113.97 (18)
C22—C21—C1	119.0 (2)	C45—N44—H44	105.8 (18)
C26—C21—C1	122.6 (2)	C46—N44—H44	107.3 (17)
C21—C22—C23	120.4 (3)	C43—N44—H44	109.1 (18)
C21—C22—H22	119.8	N44—C45—H45A	109.5
C23—C22—H22	119.8	N44—C45—H45B	109.5
C24—C23—C22	120.2 (3)	H45A—C45—H45B	109.5
C24—C23—H23	119.9	N44—C45—H45C	109.5
C22—C23—H23	119.9	H45A—C45—H45C	109.5
C25—C24—C23	119.9 (3)	H45B—C45—H45C	109.5
C25—C24—H24	120.0	N44—C46—H46A	109.5
C23—C24—H24	120.0	N44—C46—H46B	109.5
C24—C25—C26	120.4 (3)	H46A—C46—H46B	109.5
C24—C25—H25	119.8	N44—C46—H46C	109.5
C26—C25—H25	119.8	H46A—C46—H46C	109.5
C21—C26—C25	120.6 (3)	H46B—C46—H46C	109.5
C21—C26—H26	119.7	O11A—C1A—O12A	125.72 (18)
C25—C26—H26	119.7	O11A—C1A—C2A	119.27 (17)
N32—C31—C36	121.09 (19)	O12A—C1A—C2A	114.98 (16)
N32—C31—C1	115.55 (18)	O2A—C2A—C3A	108.10 (16)
C36—C31—C1	123.34 (19)	O2A—C2A—C1A	114.32 (16)
C33—N32—C31	117.84 (19)	C3A—C2A—C1A	108.68 (16)
N32—C33—C34	124.1 (2)	O2A—C2A—H2A	108.5
N32—C33—H33	118.0	C3A—C2A—H2A	108.5
C34—C33—H33	118.0	C1A—C2A—H2A	108.5
C35—C34—C33	117.8 (2)	C2A—O2A—H2A1	110 (2)
C35—C34—H34	121.1	O3A—C3A—C2A	109.67 (17)
C33—C34—H34	121.1	O3A—C3A—C4A	109.98 (16)
C34—C35—C36	119.5 (2)	C2A—C3A—C4A	109.87 (17)
C34—C35—H35	120.2	O3A—C3A—H3A	109.1
C36—C35—H35	120.2	C2A—C3A—H3A	109.1
C31—C36—C35	119.7 (2)	C4A—C3A—H3A	109.1
C31—C36—H36	120.1	C3A—O3A—H3A1	105 (2)
C35—C36—H36	120.1	O41A—C4A—O42A	126.91 (19)
C42—O41—C1	117.05 (15)	O41A—C4A—C3A	119.19 (19)
O41—C42—C43	109.65 (18)	O42A—C4A—C3A	113.88 (17)
O41—C42—H42A	109.7	C4A—O42A—H42	113.8 (17)
			
O41—C1—C21—C22	−62.2 (3)	C33—C34—C35—C36	0.4 (4)
C11—C1—C21—C22	174.4 (2)	N32—C31—C36—C35	−0.7 (4)
C31—C1—C21—C22	51.9 (3)	C1—C31—C36—C35	177.9 (2)
O41—C1—C21—C26	116.5 (2)	C34—C35—C36—C31	−0.1 (4)
C11—C1—C21—C26	−6.8 (3)	C21—C1—O41—C42	−54.2 (2)
C31—C1—C21—C26	−129.4 (2)	C11—C1—O41—C42	72.3 (2)
C26—C21—C22—C23	1.1 (4)	C31—C1—O41—C42	−170.96 (17)
C1—C21—C22—C23	179.9 (2)	C1—O41—C42—C43	−164.77 (18)
C21—C22—C23—C24	−0.4 (4)	O41—C42—C43—N44	−76.6 (2)
C22—C23—C24—C25	−0.4 (5)	C42—C43—N44—C45	−177.2 (2)
C23—C24—C25—C26	0.4 (4)	C42—C43—N44—C46	58.2 (3)
C22—C21—C26—C25	−1.1 (4)	O11A—C1A—C2A—O2A	−161.60 (19)
C1—C21—C26—C25	−179.8 (2)	O12A—C1A—C2A—O2A	20.3 (3)
C24—C25—C26—C21	0.3 (4)	O11A—C1A—C2A—C3A	77.6 (2)
O41—C1—C31—N32	179.69 (18)	O12A—C1A—C2A—C3A	−100.6 (2)
C21—C1—C31—N32	62.0 (2)	O2A—C2A—C3A—O3A	−63.9 (2)
C11—C1—C31—N32	−63.6 (2)	C1A—C2A—C3A—O3A	60.7 (2)
O41—C1—C31—C36	1.0 (3)	O2A—C2A—C3A—C4A	57.1 (2)
C21—C1—C31—C36	−116.7 (2)	C1A—C2A—C3A—C4A	−178.33 (16)
C11—C1—C31—C36	117.7 (2)	O3A—C3A—C4A—O41A	−2.5 (3)
C36—C31—N32—C33	1.1 (3)	C2A—C3A—C4A—O41A	−123.3 (2)
C1—C31—N32—C33	−177.6 (2)	O3A—C3A—C4A—O42A	179.04 (18)
C31—N32—C33—C34	−0.9 (4)	C2A—C3A—C4A—O42A	58.2 (2)
N32—C33—C34—C35	0.1 (4)		

### Hydrogen-bond geometry (Å, º)

**Table:**

*D*—H···*A*	*D*—H	H···*A*	*D*···*A*	*D*—H···*A*
C42—H42*A*···O3*A*	0.97	2.52	3.391 (3)	149
C43—H43*B*···O41*A*^i^	0.97	2.27	3.217 (3)	165
N44—H44···O12*A*	0.86 (3)	2.23 (3)	2.942 (2)	140 (2)
C46—H46*C*···O2*A*	0.96	2.51	3.084 (3)	118
O2*A*—H2*A*1···N32^ii^	0.89 (4)	1.92 (4)	2.804 (2)	171 (3)
O3*A*—H3*A*1···O41*A*	0.81 (3)	2.08 (3)	2.613 (2)	123 (3)
O42*A*—H42···O12*A*^iii^	1.12 (3)	1.33 (3)	2.4475 (18)	178 (3)

Symmetry codes: (i) *x*−1, *y*, *z*; (ii) −*x*+1, *y*−1/2, −*z*+1; (iii) *x*+1, *y*, *z*.
